# Ambient-Pressure Solvothermal Synthesis of Highly Mn-Doped Titania and Its Behavior as an Anode in Lithium-Ion Batteries

**DOI:** 10.1021/acs.inorgchem.5c02906

**Published:** 2025-09-17

**Authors:** Filip Kozłowski, Daecheol Jeong, Beichen Xiong, Geoffrey Daniel, Rafal J. Wiglusz, Fredric G. Svensson, Juanjuan Lu, Haiyan Wang, Brian M. Tackett, Gulaim A. Seisenbaeva, Vilas G. Pol, Vadim G. Kessler

**Affiliations:** † Department of Molecular Sciences, BioCenter, 8095 Swedish University of Agricultural Sciences, Almas allé 5, Box 7015, SE−75007 Uppsala, Sweden; ‡ Davidson School of Chemical Engineering, 311308 Purdue University, West Lafayette, Indiana 47907, United States; § Department of Forest Biomaterials and Technology/Wood Science, Swedish University of Agricultural Sciences, Vallvägen 9C−D, 756 51, Uppsala 750 07, Sweden; ∥ Division of Biomedical Physicochemistry, Institute of Low Temperature and Structure Research, Polish Academy of Sciences, Ul. Okolna 2, Wroclaw 50−422, Poland; ⊥ Meinig School of Biomedical Engineering, College of Engineering, Cornell University, Ithaca, New York 14853−1801, United States; # Department of Materials Science and Engineering; Solid State Physics, 8097 Uppsala University, Ångströmlaboratoriet, Lägerhyddsvägen 1, Box 35, SE−75103 Uppsala, Sweden; ¶ Materials Engineering, Purdue University, West Lafayette, Indiana 47907, United States

## Abstract

Over the past few decades, battery research has increasingly focused on titanium dioxide (TiO_2_) and manganese dioxide (MnO_2_), with TiO_2_ commonly used as an anode material and MnO_2_ as a cathode, due to their stability, abundance, and low cost. In this study, a novel TiO_2_-based material doped with high manganese (Mn) content was synthesized via a high-temperature solution-phase synthesis method using a single-source precursor for application in lithium-ion batteries (LIBs). The synthesis was conducted under controlled conditions, achieving high Mn^*n*+^ cation doping levels of up to 20–25 mol %, leading to previously unreported changes in the material’s electrochemical performance. A temperature-dependent phase transformation from anatase to rutile was observed. Samples with 5 mol %, 20 mol %, and 50 mol % Mn^*n*+^-ion doping were prepared and investigated for their structural, morphological, and electrochemical characteristics. Characterization techniques included X-ray diffraction (XRD), transmission electron microscopy (TEM), scanning electron microscopy (SEM), and cyclic voltammetry (CV). The doped materials exhibited properties distinct from those of pure TiO_2_ and pure MnO_2_, indicating effective Mn incorporation into the TiO_2_ lattice. This study highlights the potential of high-Mn-content TiO_2_-based materials as next-generation anode candidates for LIBs while also revealing the performance limitations associated with excessive Mn doping. The resulting insights into the chemistry of Ti–Mn mixed oxide anodes demonstrate the strong link between molecular precursor design and the resulting phase composition and structure. The latter is directly related to the electrochemical performance, offering a better understanding for future design and engineering of next-generation mixed oxide electrodes.

## Graphical abstract

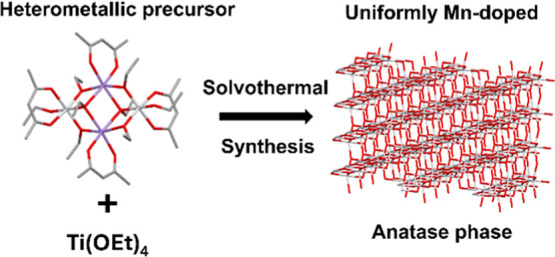

## Introduction

Current advances in battery technology increasingly target safer, more sustainable materials derived from abundant natural resources. Titanium dioxide (TiO_2_) and manganese dioxide (MnO_2_) have emerged as particularly promising candidates owing to their low cost, wide availability, and environmental friendliness. Titanium dioxide, known for its excellent chemical and electrochemical stability, has been extensively investigated as a robust anode material. For example, composite TiO_2_@C/N nanofibers have demonstrated stable LIB performance,^1,2^ while TiO_2_-encrusted MXenes and bronze-phase TiO_2_(B) have shown enhanced rate capability and cycling stability.^3,4^

Manganese dioxide, particularly in its crystalline α-phase, is a well-established high-capacity cathode in aqueous and solid-state batteries.^5^ Nano α-MnO_2_ exhibits excellent structural stability, and α-MnO_2_ coated with Mn_2_AlO_4_ delivers both high aluminum-ion storage capacity and a high discharge voltage plateau.^6^ Additionally, the electrochemical behavior of MnO_2_-based cathodes is strongly influenced by processing parameters, with both thermal and mechanical treatments shown to improve performance.^7^

The design of advanced anode materials for lithium-ion batteries (LIBs) increasingly relies on synthetic strategies capable of controlling the composition, oxidation states, and microstructure at the molecular scale.^8^ While conventional solid-state synthesis remains the industry standard, its reliance on high-temperature diffusion limits cation homogeneity and often induces phase separation, constraining electrochemical performance.^9^ In contrast, wet-chemistry-based methods, particularly sol–gel processes, offer a unique pathway to achieve atomic-level mixing of multiple cations in solution prior to oxide formation.^10^ Through controlled hydrolysis and condensation reactions, sol–gel chemistry enables fine-tuning of precursor reactivity, network formation, and crystallization, resulting in homogeneously structured mixed oxides with tailored nanostructures and functional properties.

Achieving synergy, however, requires precise atomic-scale integration of Ti and Mn within a uniform oxide lattice while controlling oxidation states, coordination geometries, and crystallinity, parameters that are difficult to regulate using traditional multi-step or high-temperature synthesis routes.^11^ Single-source precursor strategies have emerged as an effective solution, enabling cations to be pre-organized in well-defined molecular complexes prior to oxide formation.^12^

The feasibility of this approach was previously demonstrated in studies involving europium-doped BaTiO_3_ nanoparticles. The microhydrolysis of heterometallic β-diketonate alkoxides of barium and strontium, which serve as single-source precursors for perovskite oxide materials, shows their structures to arise from a thermodynamically driven self-assembly process.^13^ Building on this approach, here, we employed a high-temperature solution-phase synthesis using titanium tetraethoxide Ti­(OEt)_4_ and manganese­(II) acetylacetonate Mn­(acac)_2_ to obtain a novel material. In addition to the use of single-source precursors, a key advantage of this synthesis method was the in situ formation of the product under controlled high-temperature solution-phase synthesis conditions. Based on previous findings, the highest crystallinity of the resulting nanoparticles was achieved using aprotic ketone solvents, particularly acetophenone, thus providing the rationale for its selection in the present synthesis.^14^

Combining higher oxidation state cations with titania has already been shown to produce material with high charge capacity, as was demonstrated with titanium molybdate: TiOMoO_4_. In the latter, the molybdenum and titanium sites were structurally different. Titania occupied octahedral sites, while molybdenum ions were situated in the tetrahedral sites. This structural differentiation contributes to the material’s enhanced electrochemical performance.^15^

In this work, we present a sol–gel-based molecular precursor route employing heterometallic alkoxide–acetylacetonate complexes derived from titanium tetraethoxide (Ti­(OEt)_4_) and manganese­(II) acetylacetonate (Mn­(acac)_2_). By carefully tuning the solvent polarity, ligand coordination, and gelation kinetics, we achieve homogeneous Ti–Mn integration in the sol–gel network before crystallization. The use of aprotic ketone solvents, particularly acetophenone, promotes high crystallinity at low processing temperatures. This synthesis strategy not only ensures uniform cation distribution but also enables precise control over the particle size and phase composition, representing a significant advancement over conventional solid-state and precipitation methods.

## Experimental Section

### Materials

The following reagents were used in the present study: titanium tetraethoxide Ti­(OEt)_4_ (CAS No. 3087-36-3), manganese­(II) acetylacetonate Mn­(acac)_2_ (Sigma-Aldrich, CAS No. 24,576-3), poly­(ethylene glycol) (BioUltra, 400, Sigma-Aldrich CAS No. 25322-68-3), nitric acid HNO_3_ (65% v/v, Riedel de haen, CAS No. 7697-37-2), and acetophenone (for synthesis, Sigma-Aldrich, Cas No. 98-86-2). No unusual hazards were noted.

## Methods

### Synthesis of the Single-Source Precursor Mn_2_Ti_2_(acac)_4_(OEt)_8_

The single-source precursor was produced by dissolving the reactants Ti­(OEt)_4_ and Mn­(acac)_2_ in a 1:1 ratio in anhydrous toluene on reflux. In a typical procedure, 1 mL of Ti­(OEt)_4_ (1.09 g, 4.78 mmol) was dissolved in 20 mL of toluene, and then, 1.13 g (4.77 mmol) of Mn­(acac)_2_ powder added. The solution was subjected to reflux for 15 min with complete dissolution of the pale powder with formation of a dark brown, almost black solution. The latter was placed in a freezer at −18 °C overnight and produced dark brown rectangular needle shaped crystals with a yield of 1.73 g (78%). The identity of the product was confirmed by multiple single-crystal X-ray experiments on randomly chosen crystals.

### High-Temperature Solution-Phase Synthesis of Doped Nanoparticles with Acetophenone

Mn-doped titania nanoparticles were synthesized using titanium tetraethoxide Ti­(OEt)_4_ and manganese­(II) acetylacetonate Mn­(acac)_2_ via high-temperature solution-phase synthesis. Manganese­(II) acetylacetonate was mixed with 4 mL (fixed) of Ti­(OEt)_4_ and acetophenone to obtain final Mn doping levels of 5, 20, and 50 mol % in TiO_2_. The obtained solution was heated in an oil bath at 160 °C for 24 h. After synthesis, all samples were calcined at 500 °C, 600 °C, and 700 °C for 4 h. To confirm the structure of the obtained nanoparticles, a portion of the produced gel was also separately purified with nitric acid and analyzed.

### High-Temperature Solution-Phase Synthesis of Undoped TiO_2_

Pure titanium­(IV) oxide was synthesized under the same conditions as those described above, excluding the addition of manganese­(II) acetylacetonate. The reaction temperature and duration were identical to those used in the Mn-doped synthesis.

### Electrode Fabrication

Manganese-doped titanium oxide (Mn@TiO_2_), Super P (Timcal), and poly­(vinylidene fluoride) (PVDF, Kynar HSV–900) were mixed based on an 85:8:7 mass ratio. The mixture was dissolved in *N*–methyl pyrrolidone (NMP) before being homogenized with a Thinky mixer. The resulting slurry was cast onto copper foil and dried under vacuum at 80 °C overnight, before being punched into 12 mm circular discs. This resulted in an active mass loading of ∼2.5 mg per electrode. Pristine TiO_2_ electrodes were fabricated identically with the same 85:8:7 ratio (TiO_2_: Super P: PVDF). Graphite electrodes were prepared by using mesocarbon microbeads (MCMB, MSE), Super P, and PVDF at an 8:1:1 mass ratio. Slurries were processed as above, cast on copper foil, dried at 80 °C under a vacuum, and punched into 12 mm disks. LiFePO_4_ (LFP, MSE) cathodes were prepared with an 8:1:1 mass ratio of LFP:Super P:PVDF, dispersed in NMP, homogenized, and cast onto aluminum foil. After being dried at 80 °C under vacuum, the electrodes were punched into 12 mm disks.

## Characterization

### Cyclic Voltammetry (CV)–Electrochemical Characterization

CR2032 coin cells were assembled with the 12 mm Mn@TiO_2_ electrodes as the cathode, 14 mm lithium-coated copper foil (MSE supplies) as the anode, and 16 mm Celgard separators (polypropylene membrane, Celgard 2500). Each cell was filled with 25 μL of electrolyte. For Mn@TiO_2_ || LFP and TiO_2_ || LFP full cells, 14 mm TiO_2_ or Mn@TiO_2_ electrodes were paired with 12 mm LFP cathodes, and the N/P capacity ratio was controlled at 1 to ensure a balanced lithium inventory between the anode and cathode.

Cyclic voltammetry (CV) was performed by using a potentiostat (BioLogic VMP 300). The CV tests for the TiO_2_ || Li cells and 20%Mn@TiO_2_ || Li cells were conducted in the potential range between 1.1 and 3.0 V (vs Li/Li^+^) at 0.1 mV/s scan rate. For graphite || Li cells, CV measurements were carried out between 0.01 and 1.5 V­(vs Li/Li^+^) at a scan rate of 0.1 mV s^–1^.

Galvanostatic charge/discharge measurement was carried out using a battery-testing system (Arbin BT-2000). TiO_2_ || Li and Mn@TiO_2_ || Li half-cells were cycled in the voltage range of 1.1–3.0 V (vs Li/Li^+^) at both room temperature (25 °C) and elevated temperature (50 °C). Graphite || Li half-cells were cycled from 0.01 to 1.5 V (vs Li/Li^+^), and full cells (Mn@TiO_2_ || LFP and TiO_2_ || LFP) were cycled from 0.1 to 3.0 V, both at room temperature only. All cells underwent five formation cycles at 20 mA g^–1^ within their respective voltage windows at 25 °C prior to subsequent tests. For long-term cycling, TiO_2_-based half-cells were subsequently cycled at 50 mA g^–1^. For rate capability studies, cells were cycled seven times at current densities of 20, 50, 100, 200, and 500 mA g^–1^ within the appropriate voltage window.

### X-ray Photoelectron Spectroscopy (XPS)

X-ray photoelectron spectroscopy (XPS) measurements were performed with a Quantera II Scanning XPS Microscope from Physical Electronics equipped with an Al Kα source. A low-energy flood gun, set at 1.0 V and 20.0 μA, was employed for charge compensation. For the survey spectra, a pass energy of 224 eV and a resolution of 0.8 eV were used, and for the high-resolution spectra, a pass energy of 55 eV and 0.1 eV resolution were employed. Sample powders were suspended in water and dropped onto clean glass slides with subsequent evaporation of the solvent under a desktop lamp. The binding energy was calibrated against the C 1s peak at 284.8 eV from adventitious carbon. Data were treated and analyzed using the CASA XPS software.^16^ The spectra were smoothed using a Savitzky–Golay algorithm with a 9-point window.

### Scanning Electron Microscopy (SEM)

Scanning electron microscopy observations were carried out using a Hitachi FlexSEM 1000 II at an accelerating voltage of 5.00 kV, a working distance of 5.5 mm, and a spot size of 20 μm. Samples were prepared on carbon tape from suspensions.

### Energy-Dispersive X-ray Spectroscopy (EDS)

Energy-dispersive X-ray spectroscopy observations were conducted using X–Stream–2/micsF+ (Oxford Instruments, Oxford, UK) at an acceleration voltage of 15.00 kV, a spot size of 50 mm, and 1 mm working distance.

### Atomic Force Microscopy (AFM)

The surface morphologies of the material and nanoparticles were characterized with an atomic force microscope (FastScan Bio, JPK) with a Nanoscope V controller in the ScanAsyst mode using an AFM probe (Silicon tip, *f*_0_:400 kHz, k:4 N/m, Tip radius: 5 nm nominally) and a scan rate of 1–3 Hz. Gwyddion 2.56 software was used for data processing.

### X-ray Diffraction (XRD)

The X-ray powder diffraction patterns were recorded for the gel-like materials put into a glass capillary (Lindeman tube) on the multipurpose Bruker D8 Quest ECO diffractometer operating with Mo Kα radiation (λ = 0.71073 Å). Bruker Apex–IV and Eva software were used for integration and data treatment.

### Single-Crystal X-ray Diffraction Study of the Precursor Compound

Single-crystal X-ray diffraction data were collected on a Bruker D8 Quest ECO diffractometer equipped with Mo Kα radiation (λ = 0.71073 Å). A total of 2424 frames were recorded over a total exposure time of 2.02 h. Data integration was performed using the Bruker SAINT software package employing a narrow-frame algorithm.

Integration within a monoclinic unit cell yielded 20,443 reflections to a maximum θ angle of 25.02° (corresponding to a resolution of 0.84 Å), of which 4329 were independent (redundancy = 4.722; completeness = 98.4%; Rint = 5.09%, Rsig = 3.69%). Of these, 3132 reflections (72.35%) had intensities greater than 2σ­(F^2^). Final unit cell parameters were determined from the refinement of XYZ centroids of 5805 reflections with I > 20σ­(I) and 5.800° < 2θ < 46.81°, giving *a* = 11.938(5) Å, *b* = 14.846(6) Å, *c* = 15.120(6) Å, β = 111.710(6)°, and *V* = 2489.7(17) Å^3^. Absorption corrections were applied using the multiscan method implemented in SADABS. The ratio of minimum to maximum apparent transmission was 0.865, while the calculated transmission coefficients based on crystal size ranged from 0.7950 to 0.9340. The structure was solved and refined using the Bruker SHELXTL software suite. The crystal was determined to belong to the monoclinic space group P1 21/n 1, with *Z* = 4 for the molecular formula C_16_H_30_MnO_8_Ti. Full-matrix least-squares refinement on F^2^ with 288 variables converged at R1 = 5.79% [for I > 2σ­(F^2^)] and w*R*2 = 15.96% (for all data). The goodness-of-fit on F^2^ was 1.086.

The final difference Fourier map showed a maximum electron density peak of 0.620 e^–^/Å^3^ and a minimum of −0.550 e^–^/Å^3^, with an RMS deviation of 0.052 e^–^/Å^3^. The calculated density was 1.209 g·cm^–3^, with F(000) = 948 electrons.

Crystallographic data for the structural analysis have been deposited with the Cambridge Crystallographic Data Centre (CCDC), deposition number 2464817, and are available free of charge at https://www.cam.ac.uk.

### Transmission Electron Microscopy (TEM)

The diffraction patterns, transmission electron microscopy (TEM) and scanning transmission electron microscopy (STEM) images, and energy-dispersive spectra (EDS) mapping for the pure TiO_2_ and Mn-doped TiO_2_ nanoparticles were obtained using a FEI ThermoFisher TEM TALOS 200X operated at 200 kV.

## Results and Discussion

In order to produce highly Mn-doped titania, we incorporated highly charged Mn­(III)/Mn­(IV) cations, displaying ionic radii of 0.65 and 0.53 Å, respectively, comparable to those of Ti­(IV), 0.605 Å, into the anatase structure. Developing this strategy and after successfully producing a Ti–Mo precursor,^15^ we investigated the possibility to synthesize a heterometallic alkoxide complex with the highest possible (i.e., 1:1) ratio that could be used with the excess of titanium alkoxide in subsequent solvothermal synthesis to access a complex oxide with the desired composition. We exploited the construction principles of the heterometallic alkoxide complexes. This involves the known stability of the M_4_O_16_ core structure when using linear chain (primary) alkoxide ligands and the fact that the acetylacetonate ligand is structurally equivalent to two ethoxide groups.^17^ The required metal-to-donor atom ratio was available by simply combining (anhydrous) manganese acetylacetonate with titanium ethoxide in a nondonor solvent like toluene

$${2Mn\left(acac\right)}_{2}+{2Ti\left(OEt\right)}_{4}\to {Mn}_{2}{Ti}_{2}{\left(acac\right)}_{4}{\left(OEt\right)}_{8}$$

The reaction proceeded almost quantitatively with 78% yield in direct low-temperature recrystallization with the possibility to recover the rest of the same material by subsequent evaporation of the solvent in vacuum.

The structure of the produced compound was of the so-called titanium ethoxide type with a M_4_O_16_ core (see Figure 1). The compound formed dense hexagonal packing or a trioctahedral motif typical of layered oxide minerals.^18^ The molecular structure was clearly derived from Mn­(II) atoms, displaying apparent distinction between the longer Mn–O bonds, especially toward alkoxide ligands, Mn(1)–O(5) 2.259(3) and 2.304(2) Å for triply bridging ethoxide ligands and Mn(1)–O(1) 2.179(3) and Mn(1)–O(6) 2.182(3) Å for doubly bridging ligands. The shortest bonds for manganese cations were observed at the stronger charged (less basic) acac ligand, Mn(1)–O(7) 2.116(3) Å and Mn(1)–O(8) 2.095(3) Å. The coordination geometry of Ti atoms indicates a much smaller atomic radius and a stronger covalent contribution: Ti(1)–O(4) 1.786(3) Å terminal—a length comparable to that of Ti = O double bonds as in, for example, [(^i^PrOH)­BaTiO­(O^i^Pr)_4_]_4_.^19^ The distances to doubly bridging oxygen atoms Ti(1)–O(1) 1.888(3) and Ti(1)–O(6) 1.897(3) Å and to triply bridging oxygen atoms Ti(1)–O(5) 2.053(2) Å were also somewhat shorter than those commonly observed. The titanium–oxygen distances in the attachment of the acac ligand were almost 0.1 Å shorter than otherwise revealed in an octahedral coordination of Ti­(IV), Ti(1)–O(3) 2.036(3) and Ti(1)–O(2) 2.043(3) Å.^20^ The observed bond distances permit the formulation of the obtained single-source precursor as a close ion pair, [Mn­(acac)]^+^_2_[Ti­(acac)­(OEt)_4_]^−^_2_. It is worth noting that in spite of a distinct charge distribution in the structure, the produced complex demonstrates clear charge-transfer behavior, manifested by its dark brown, almost black color. It indicates the apparent oxidation of Mn­(II) in this environment, opening for high doping into the anatase structure on further treatment in a non-deoxygenated environment.

**Figure 1:**
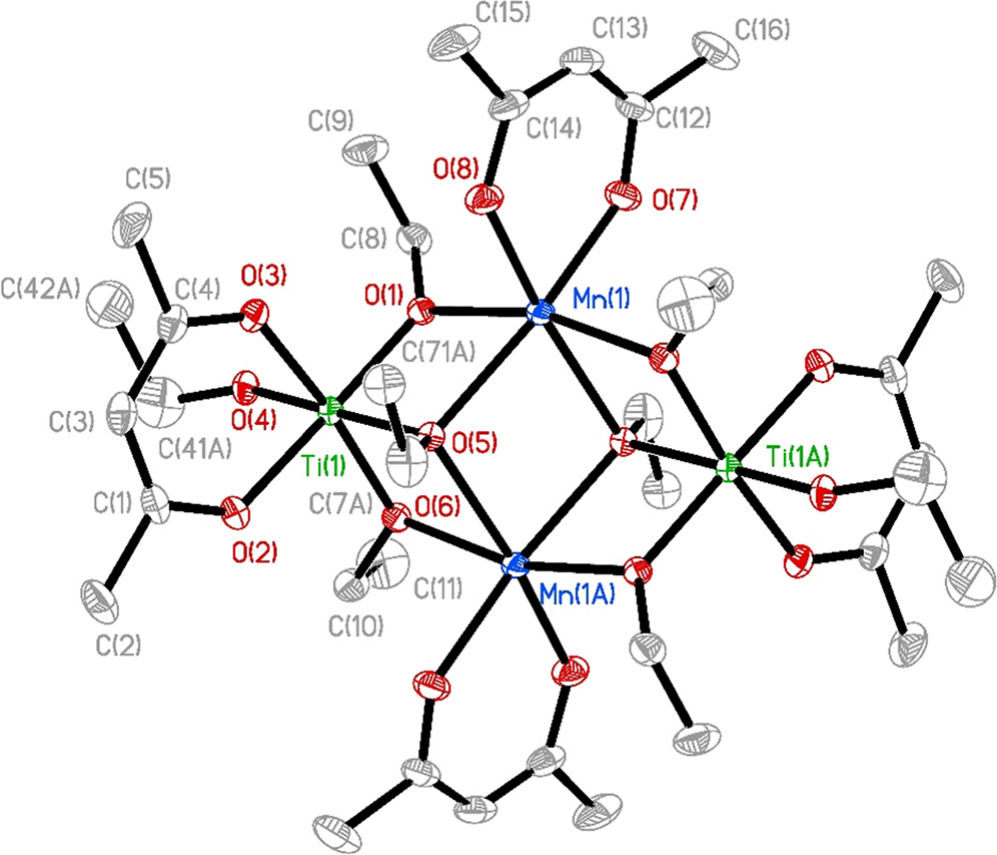
Molecular structure of the Mn_2_Ti_2_(acac)_4_(OEt)_8_ compound.

Synthesis of the materials was carried out by high-temperature solution-phase synthesis treatment in acetophenone medium. This follows the trend for the formation of relatively highly crystalline phases on solution decomposition in refluxing acetophenone (*T*_b_ = 202 °C), eliminating the need for an autoclave for solvothermal synthesis.^21^ Synthesis was performed by dissolving anhydrous Mn­(acac)_2_ and Ti­(OEt)_4_ in cation ratios ranging from 2 to 50 mol % in the chosen solvent and subjecting the obtained dark solution (indicating the formation of the mixed-metal precursor as an intermediate, see Figure S4 in the Supporting Information) on the way to the oxide phase.

The SEM, AFM, TEM, and X-ray diffraction analyses collectively indicate that the synthesized materials remain homogeneous and monophasic up to approximately 20 mol % manganese doping (Figures 2 and 3). Beyond this concentration, the formation of manganese oxide phases increases, although these secondary phases are not observed under SEM. The use of an aprotic ketone solvent, specifically acetophenone, enabled the synthesis of materials exhibiting remarkably high crystallinity (see also Figures S1–S4 in the Supporting Information). Thermal treatment induced a temperature-dependent phase transformation. Samples calcined at 700 °C showed a transition from the anatase to the rutile phase. At 600 °C, the samples contained a mixture of anatase and rutile phases, whereas calcination at 500 °C predominantly yielded the anatase phase. These observations align well with the previously reported behavior for titania-based materials.

**Figure 2:**
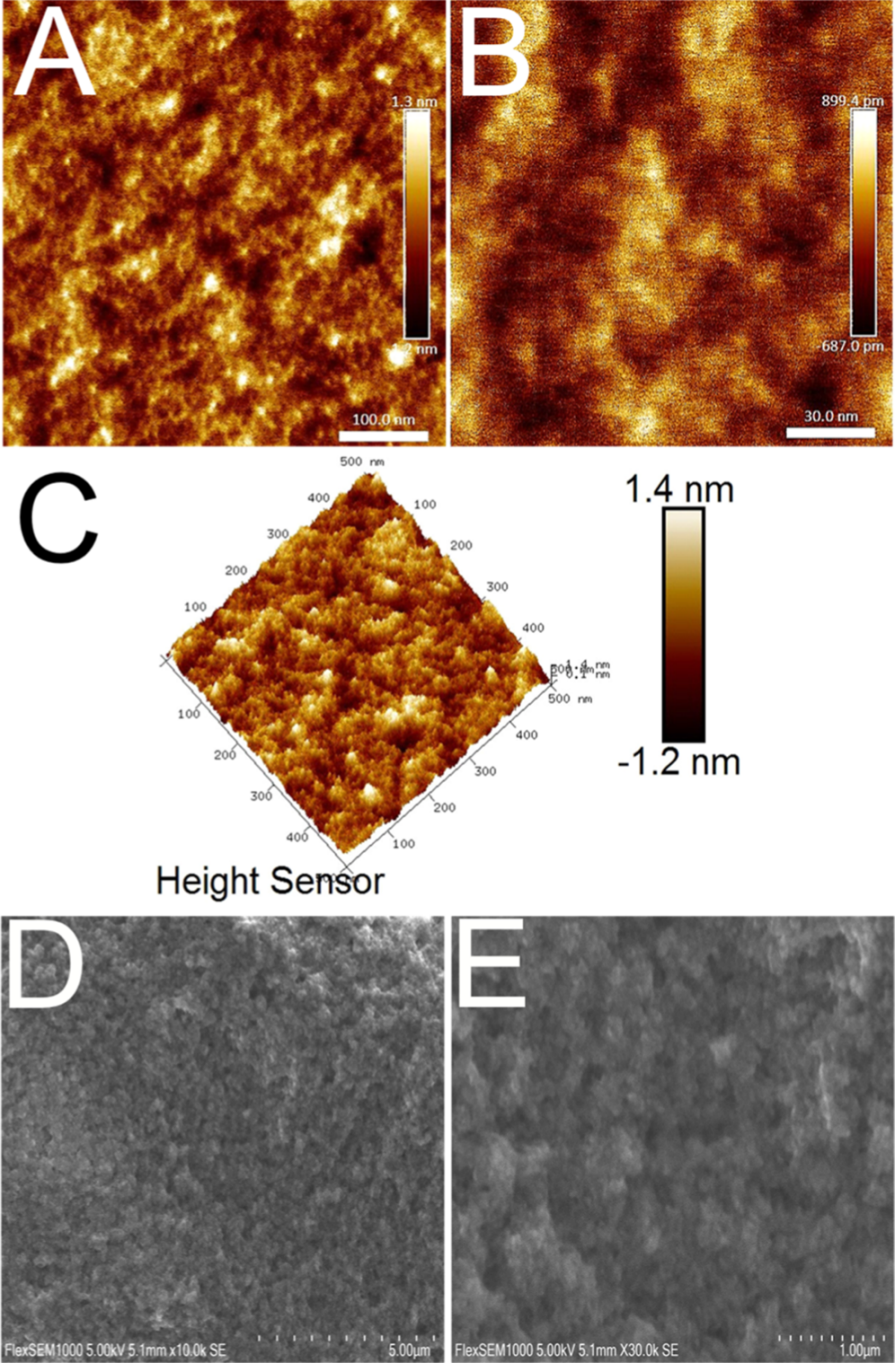
AFM micrographs of high-temperature solution-phase synthesis products at different magnifications, displaying highly crystalline nanoparticles (A–C). SEM micrographs showing the material after thermal treatment. The aggregated structure of the nanoparticles can be seen (D,E).

**Figure 3:**
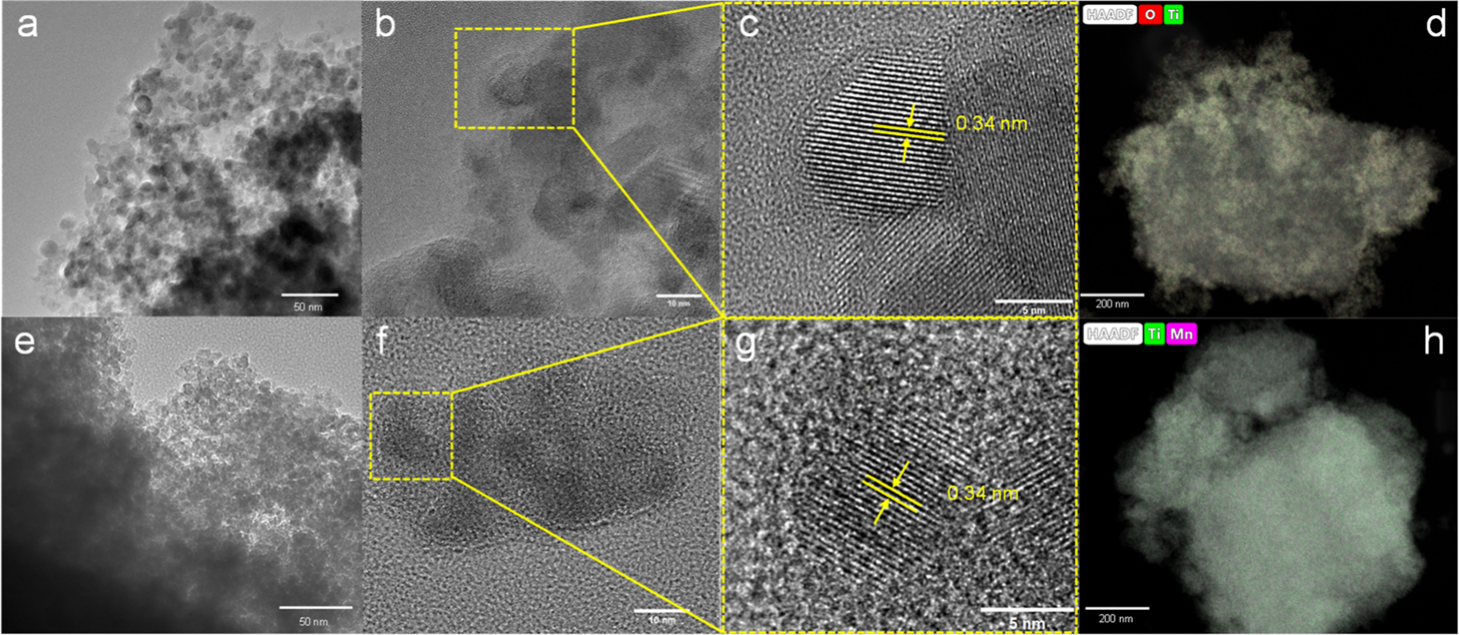
Transmission electron microscopy (TEM) micrographs at different magnifications. (a–d) Pristine TiO_2_ nanoparticles. (e–h) 20 mol % Mn-doped TiO_2_ nanoparticles crystals with high crystallinity.

All samples as prepared and after annealing at 500 °C displayed a single-phase anatase pattern in the X-ray powder diffractograms (Figure 4), except for those with 50% Mn doping, where a separate phase of Mn_3_O_4_ hausmannite was clearly observed as an admixture along with a rutile phase of TiO_2_ (see Tab. TS1 in the Supporting Information).

**Figure 4:**
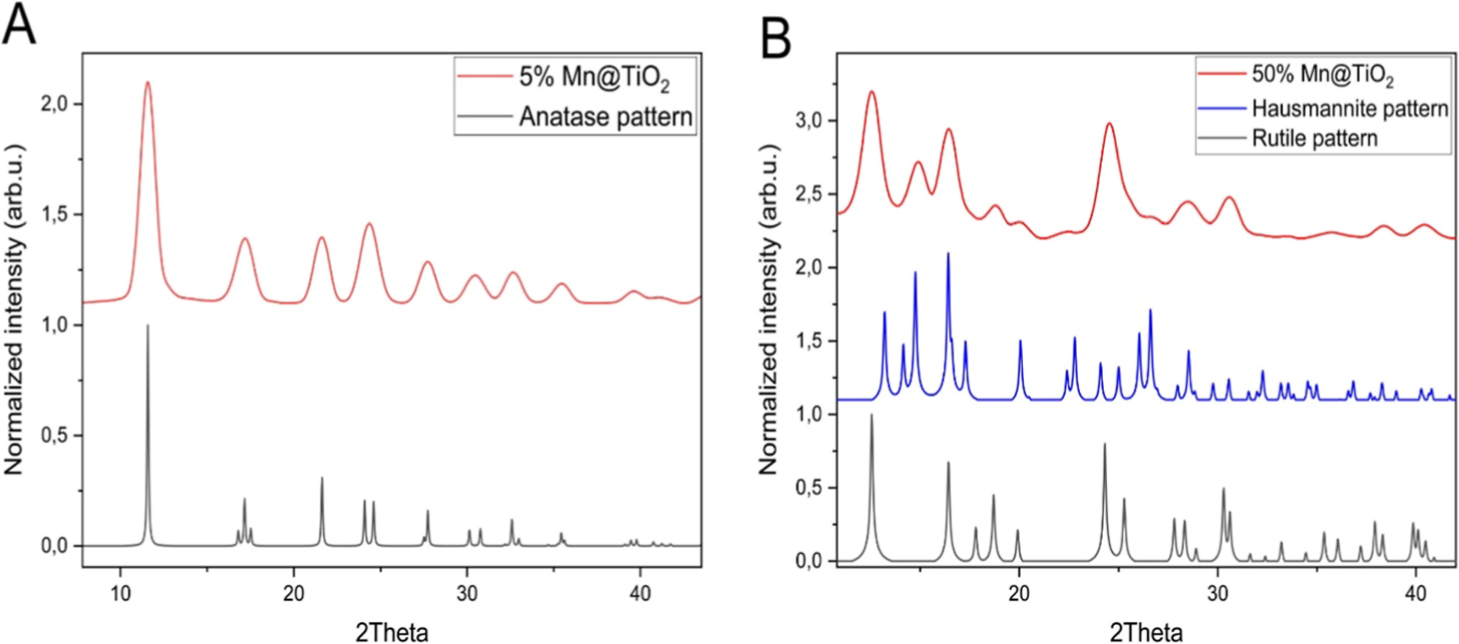
XRD patterns of 5% (A) and 50% (B) Mn-doped titania annealed at 500 °C for 1 h. Reference peak positions are indicated for anatase (JCPDS 21-1272),^22^ rutile (JCPDS 21-1276),^23^ and hausmannite (JCPDS 24-0734).^24^ Reference patterns were calculated from the respective JCPDS data.

The surface chemistry of the two manganese-doped titania was investigated by XPS to gain a deeper insight into the oxidation state(s) of the manganese. Application of XPS as a bulk method was adequate in this case since the particle size of the obtained materials according to TEM (see Figure 3) was in the interval 7–10 nm and the depth of XPS penetration commonly estimated to be 2–3 nm was deep enough for the small particles studied. Considerable difference was observed between the lower and higher manganese-doped titania concentrations (Figure 5). From the survey spectra, the presence of titanium, manganese, and oxygen could be confirmed. It is evident that the sample with 20% manganese doping contains more manganese (Figure 5b), while the manganese signal is much weaker for the 5% doped material.

**Figure 5:**
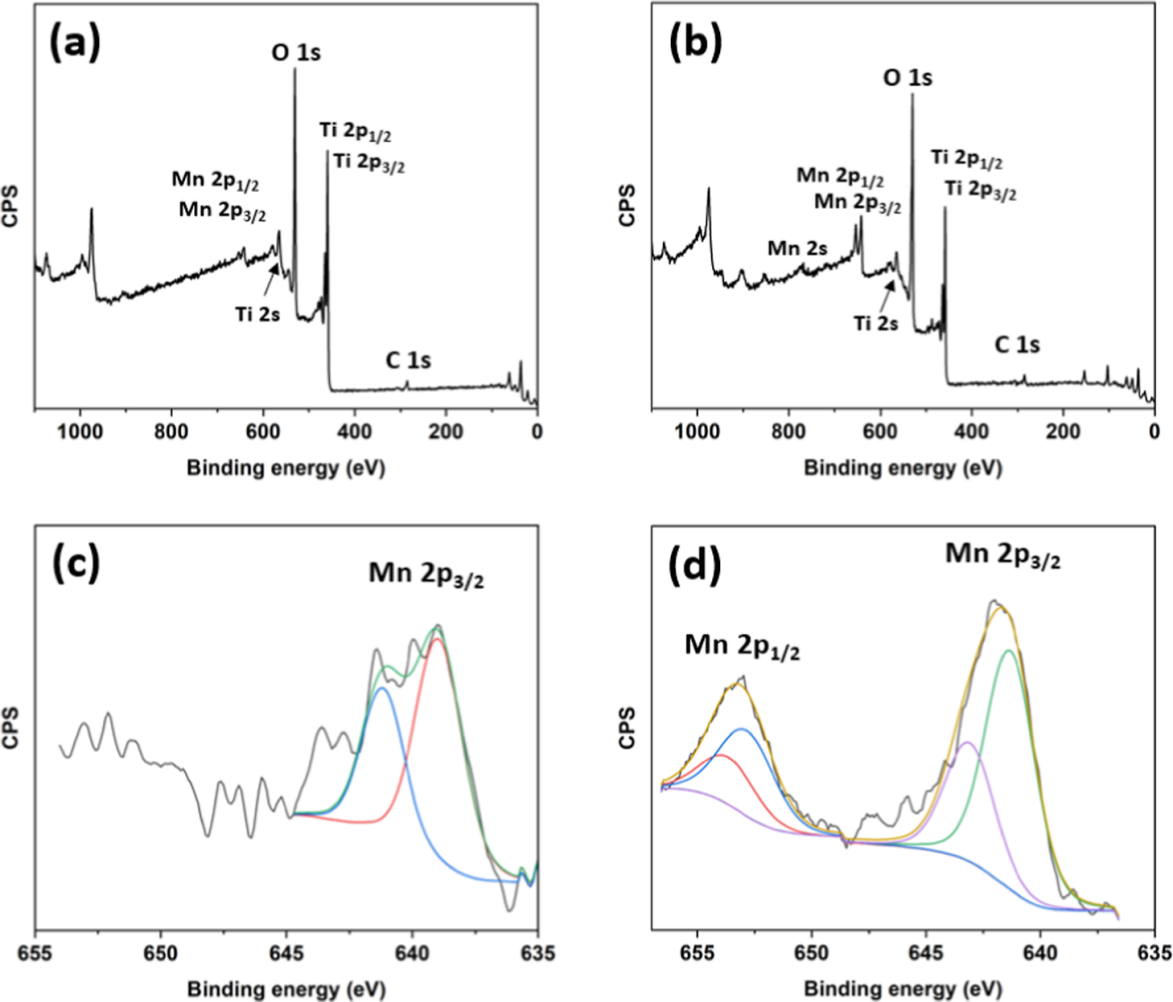
XPS survey spectra of (a) 5% Mn@TiO_2_ and (b) 20% Mn@TiO_2_. (c) High-resolution XPS spectrum of the 5% Mn@TiO_2_ sample. Only the 2p_3/2_ peak could be resolved. Deconvolution indicates the presence of Mn^3+^ alongside lower oxidation states, specifically Mn^0^ and possible Mn^2+^. (d) High-resolution XPS spectrum of the 20% Mn@TiO_2_ sample. Deconvolution of the 2p_1/2_ and 2p_3/2_ peaks revealed the coexistence of Mn^3+^ and Mn^4+^ oxidation states, indicating a mixed valence state on the particle surface.

The high-resolution XPS spectrum for 5% Mn@TiO_2_ was weak, and only the 2p_3/2_ peak could be reasonably resolved. It was deconvoluted into two components, revealing the presence of manganese in the 3+ oxidation state (641.2 eV) and metallic manganese (639.0 eV).

For the 20% Mn@TiO_2_ sample, the 2p_1/2_ and 2p_3/2_ peaks could be deconvoluted into two major components indicative of Mn^3+^ (653.1 and 641.4 eV) and Mn^4+^ (653.9 and 643.2 eV) with a characteristic spin-orbit splitting of ca. 11.7 eV.

### Electrochemical Characterization

To study the effect of different amounts of Mn on electrochemical performance of the Mn@TiO_2_ electrodes, long-term cycling was conducted with the commercial 1 M LiPF_6_ in 1:1 vol % EC/DEC electrolyte (Figure 6a). The cells were cycled at 50 mA/g after five formation cycles at 20 mA/g. Among the three electrodes, 20% Mn@TiO_2_ exhibited the highest reversible capacity of 43 mAh/g, along with an average Coulombic efficiency (CE) of 99.6% and a capacity retention of 88.9% after 90 cycles. 5% Mn@TiO_2_ displays a similar 92.7% capacity retention, but with a lower capacity of 27 mAh/g and an average CE of 94%, with a rapid decay of CE taking place around cycle 30. The 50% Mn@TiO_2_ sample initially showed a higher capacity to the 20% electrode during the formation cycles but quickly dropped to 35 mAh/g at 50 mA/g. Its unusually high average CE of 100.4% suggests the presence of parasitic redox processes, such as Mn dissolution and structural degradation, which are known to occur in Mn-containing systems due to disproportionation and irreversible side reactions.^16^

**Figure 6:**
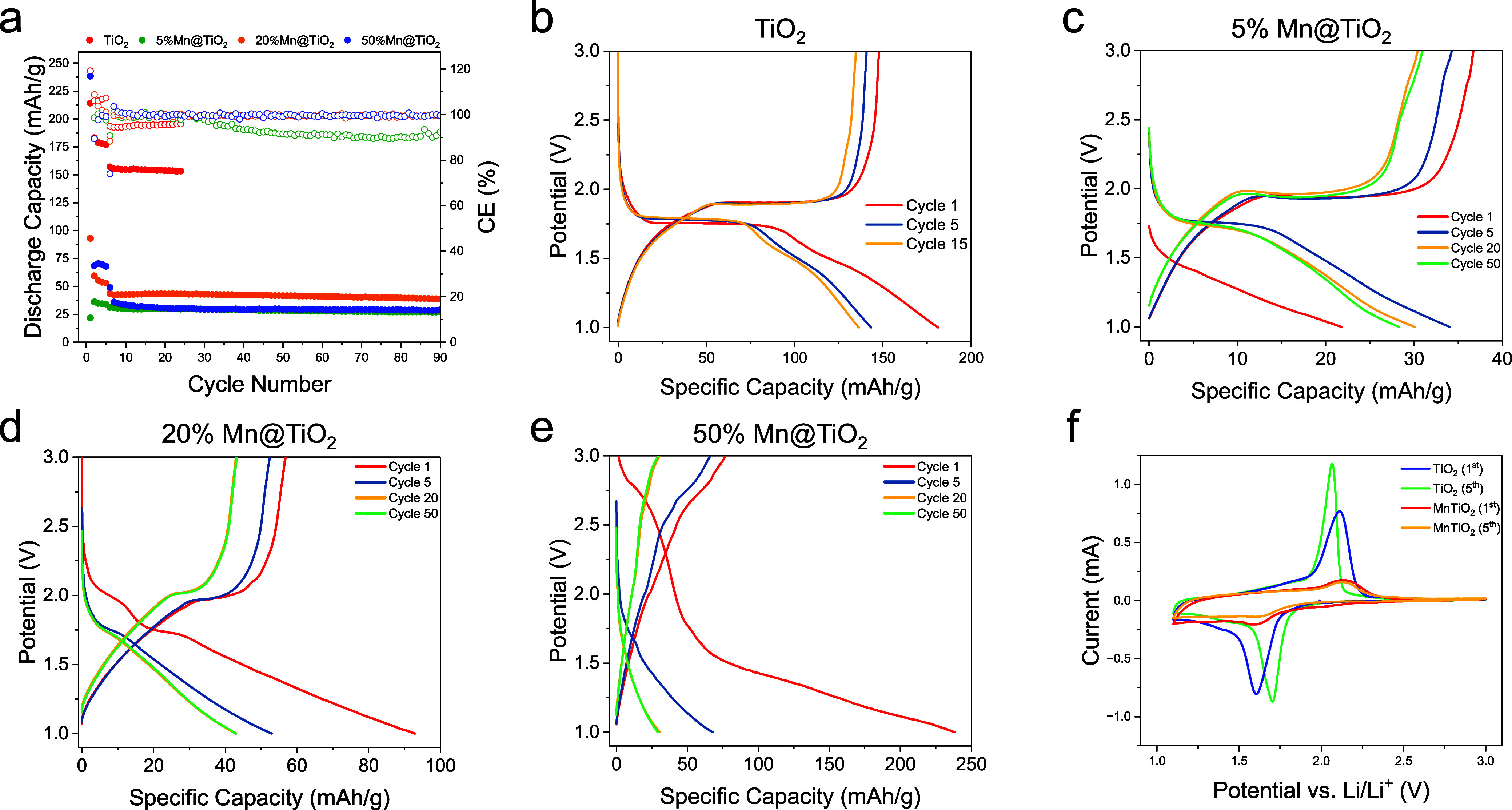
Electrochemical performance of TiO_2_ and Mn@TiO_2_ electrodes. (a) Long-term cycling result of the TiO_2_ || Li cell and Mn@TiO_2_ || Li cell at room temperature at 50 mA g^–1^ after five formation cycles at 20 mA/g. (b–e) Voltage–capacity profiles at various cycles for (b) pristine TiO_2_, (c) 5% Mn@TiO_2_, (d) 20% Mn@TiO_2_, and (e) 50% Mn@TiO_2_. (f) Cyclic voltammetry (CV) of TiO_2_ and 20% Mn@TiO_2_ electrodes at 0.1 mV/s.

Voltage profiles (Figure 6b–e) further clarify the evolution of reaction mechanisms with increasing Mn content. The pristine TiO_2_ electrode exhibits a well-defined discharge plateau around 1.75 V and a sloping profile below 1.7 V, consistent with Li^+^ intercalation into the TiO_2_ lattice and the Ti^4+^/Ti^3+^ redox couple (Figure 6b). A similar profile is observed in the 5% Mn@TiO_2_ electrode (Figure 6c), suggesting that at low doping levels, the electrochemical behavior remains dominated by the TiO_2_ host structure.^25,26^ In the 20% Mn@TiO_2_ electrode, a diminished 1.75 V intercalation plateau and a shift in the capacity contribution toward voltages below 1.7 V suggest that Mn incorporation modifies the electrochemical reaction pathway. Notably, cyclic voltammetry (CV) shows that while the overall redox profile remains similar to that of pristine TiO_2_, the current response becomes significantly lower with Mn doping (Figure 6d,f). This decrease in current indicates that Mn substitution may suppress Ti^4+^/Ti^3+^ redox activity by blocking intercalation pathways or altering the electronic environment, thereby reducing the total number of active sites for lithium insertion. Since Mn redox transitions generally occur above 2.0 V vs Li^+^/Li,^27^ the sub-1.7 V capacity observed here likely originates from alternative mechanisms.

In the 50% Mn@TiO_2_ electrode (Figure 6e), a distinct discharge plateau around 2.7 V was observed during the early cycles, which may be attributed to Mn^4+^/Mn^3+^ redox activity, as similarly reported in MnO_2_-based electrodes.^27–30^ However, this plateau rapidly diminishes with cycling, evolving into a broad sloping profile characteristic of conversion-type reactions and structural degradation. This suggests that while high Mn content activates Mn redox at early stages, it destabilizes the electrode structure during prolonged cycling, leading to poorer capacity retention and less defined electrochemical behavior.

To investigate the effect of Mn doping on the rate capability of TiO_2_-based electrodes, Mn@TiO_2_||Li half-cells using the same commercial 1 M LiPF_6_ in EC/DEC electrolyte were subjected to a progressive rate test at room temperature (Figure 7a–d). The current density was increased stepwise from 20 to 500 mA/g and then returned to 20 mA/g to evaluate the recovery. As the current increased, all three Mn@TiO_2_ compositions (5%, 20%, and 50%) exhibited typical polarization effects, including shortened and shifted voltage plateaus. Notably, the voltage–capacity curves at the end of the test (after returning to 20 mA/g) closely matched their original profiles, indicating that the electrode structures remained intact and electrochemically stable throughout high-rate cycling. This recovery behavior confirms that Mn doping does not severely degrade the structural integrity of the TiO_2_ host during fast cycling.

**Figure 7:**
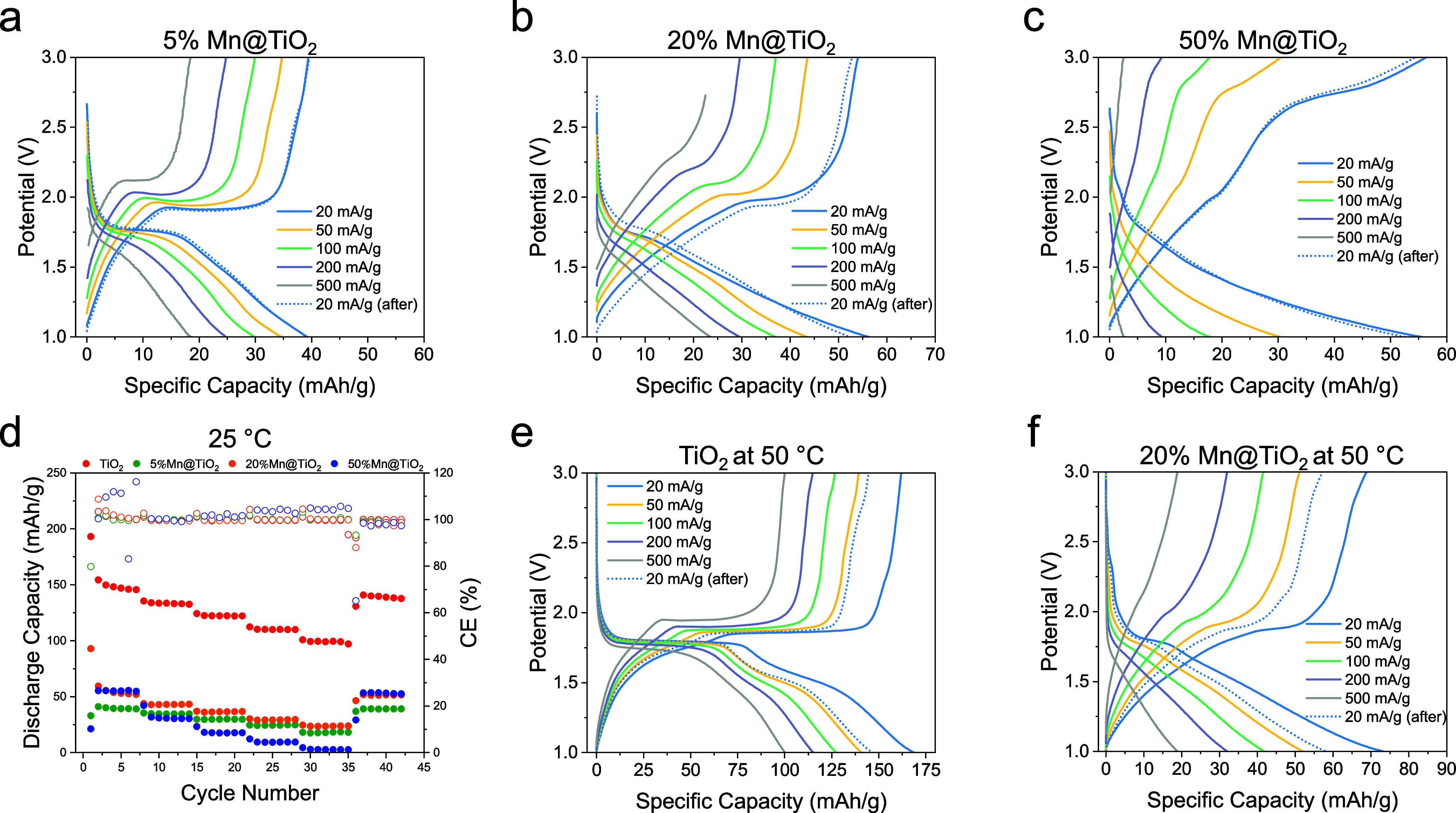
Rate performance of Mn@TiO_2_ and TiO_2_ electrodes. (a–c) Voltage–capacity profiles of (a) 5%, (b) 20%, and (c) 50% Mn@TiO_2_ electrodes at room temperature. (d) Discharge capacity and Coulombic efficiency (CE) of TiO_2_ and Mn@TiO_2_ electrodes over rate cycling at room temperature. (e–f) Voltage–capacity profiles at 50 °C for (e) pristine TiO_2_ and (f) 20% Mn@TiO_2_.

Among the three electrodes, 20% Mn@TiO_2_ demonstrated the best trade-off between capacity and rate performance. It delivered a relatively high capacity even at 500 mA/g and quickly recovered when the current was reduced. In contrast, the 50% Mn@TiO_2_ sample exhibited severe polarization (Figure 7c), evident from its rapidly distorted voltage profiles and suppressed capacity under high-rate conditions. These results suggest that excessive Mn doping introduces kinetic limitations or conversion-type behavior that degrades high-rate performance (Figure 7d).

To further probe the effect of temperature on rate performance, a similar rate test was conducted at 50 °C for the pristine TiO_2_ and 20% Mn@TiO_2_ electrodes (Figure 7e,f). Both electrodes exhibited enhanced capacities at elevated temperatures due to accelerated Li^+^ transport kinetics. In particular, the 20% Mn@TiO_2_ sample showed a noticeable improvement in high-rate capacity and less pronounced polarization compared to its room-temperature performance. However, while elevated temperature helped boost capacity, it may also intensify side reactions or structural degradation over prolonged cycling, especially in Mn-containing systems.

For comparison with the conventional anode material, the electrochemical performance of commercial graphite was also evaluated (Figure S5). Graphite exhibits the expected irreversible capacity loss during the initial cycle due to solid–electrolyte interphase (SEI) formation, followed by highly reversible lithiation/delithiation behavior (Figure S5a–c).^31^ Although graphite provides significantly higher reversible capacity, its performance deteriorates at elevated current densities, showing pronounced polarization, reduced capacity retention, and unstable cycling (Figure S5d,e). By contrast, the Mn@TiO_2_ electrodes retain a larger fraction of their capacity under high-rate conditions (Figure 7d), with a stable recovery when the current density is reduced. These results indicate that while Mn@TiO_2_ does not compete with graphite in terms of specific capacity, it offers a superior rate capability.

To further evaluate the practical applicability of the Mn-doped electrodes, full cells were assembled by using a commercial LiFePO_4_ (LFP) cathode paired with TiO_2_ or 20% Mn@TiO_2_ anodes (Figure 8a,b). Consistent with the half-cell results (Figure 6c–e), the Mn@TiO_2_ electrode undergoes a pronounced irreversible capacity loss during the first cycle, which can be attributed to structural and electrochemical changes induced by Mn incorporation. Following this initial process, however, the LFP||20% Mn@TiO_2_ full cell exhibits stable cycling. Although its reversible capacity does not exceed that of the pristine TiO_2_ anode, the stable operation highlights the improved cycling stability of 20% Mn@ TiO_2_ during extended testing.

**Figure 8:**
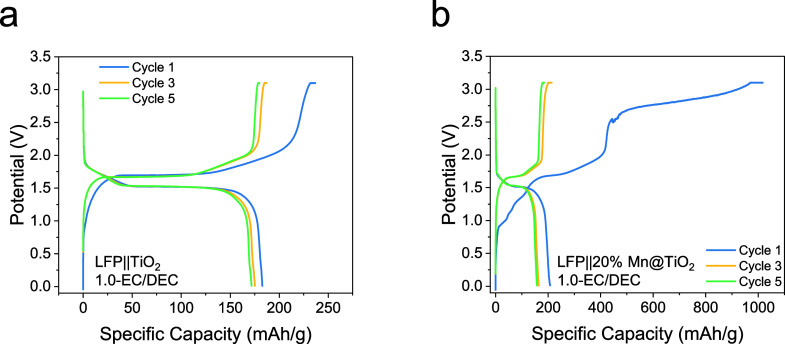
Cycling performance of full cells at RT. (a) LFP||TiO_2_. (b) LFP||20% Mn@TiO_2_.

Ex situ XRD analysis was performed on TiO_2_ and 20% Mn@ TiO_2_ electrodes before and after extended cycling to assess structural integrity (Figure S6). The diffraction patterns of the cycled electrodes are essentially identical to those of their pristine counterparts with no detectable peak shifts, broadening, or emergence of secondary reflections. The persistence of the anatase phase indicates that the parent crystal structure is maintained throughout repeated Li^+^ insertion/extraction and that neither conversion reactions nor bulk phase transitions occur within the resolution of laboratory XRD.

In summary, while high Mn^*n*+^ doping (20 mol %) slightly enhances the reversible capacity and rate performance compared to lower or extremally high doping levels, the overall electrochemical advantage of Mn incorporation into TiO_2_ remains limited. The pristine TiO_2_ electrode consistently outperforms Mn^*n*+^-doped samples in both long-term cycling and high-rate capability, especially at elevated temperature. Although 20% Mn@TiO_2_ shows a brief benefit in terms of rate stability and capacity recovery, its lower CV current response and capacity below 1.7 V suggest that Mn substitution may suppress Ti^4+^/Ti^3+^ intercalation activity, possibly by blocking lithium insertion pathways or introducing structural disorder. High Mn content (50%) triggers Mn redox activity initially but rapidly transitions to conversion-type behavior with significant polarization and capacity decay. Overall, Mn doping introduces both kinetic and structural trade-offs that outweigh its redox contribution under the conditions studied and thus does not confer a clear electrochemical advantage in TiO_2_-based lithium-ion batteries.

This behavior contrasts markedly with that observed in titanium molybdate systems, where reduction processes occurred primarily at structurally distinct Mo (VI) sites. In that case, the redox activity of molybdenum did not impede lithium-ion diffusion, likely due to the spatial separation of redox centers and diffusion pathways.

## Conclusions

The application of a single-source precursor approach in high-temperature solution-phase synthesis enabled the successful preparation of highly Mn-doped TiO_2_. The resulting material is a nanocrystalline powder with a uniform particle size below 10 nm and a remarkably homogeneous chemical composition. Notably, the anatase structure is preserved even at unusually high Mn doping levels, with the limit approximately 20 mol %, with manganese evenly distributed throughout the lattice. XPS analysis revealed an increase in the oxidation state of surface Mn species with increasing doping levels. At 20% Mn content, both Mn^4+^ and Mn^3+^ ions were observed, whereas at 5 mol % Mn doping, a mixture of Mn^2+^ and Mn^3+^ ions was detected. Contrary to expectations that manganese doping would enhance lithium storage capacity, the doped materials exhibited reduced capacity and cycling stability compared with undoped TiO_2_ or pure MnO_2_ phases. This may be caused by local stabilization of oxidation states in the TiO_2_ structure incorporating stable oxidized manganese centers and hindering lithium-ion diffusion. Additionally, the reduction of Mn^4+^ without alteration of the Ti oxidation state may further prevent effective lithium intercalation, limiting the electrochemical performance of the doped system.

## Supplementary Material



## Notes

Details of Rietveld refinement for 2 mol % Mn-doped TiO_2_, high-resolution TEM images of 20% Mn-doped and undoped solvothermal produced TiO_2_, simulated powder X-ray diffraction (PXRD) pattern, calculated from the single-crystal structure and measured from the bulk precipitate from the single-source precursor, electrochemical characterization includes cyclic voltammetry (CV) profiles of graphite, comparative XRD patterns before and after cycling for TiO_2_ and 20% Mn@TiO_2_ samples, and a comprehensive summary table of X-ray powder diffraction data for all synthesized samples (PDF)
