# Supplementary material for: Ambient-Pressure Solvothermal Synthesis of Highly Mn-Doped Titania and Its Behavior as an Anode in Lithium-Ion Batteries
Source: Inorg Chem. 2025 Sep 17;64(38):19296–305. doi: 10.1021/acs.inorgchem.5c02906 (PMC12486198; doi:10.1021/acs.inorgchem.5c02906)
Supplement: Supplementary file 1 [file ic5c02906_si_001.pdf]

Supporting information

Ambient pressure solvothermal synthesis of highly Mn–doped titania and its behaviour as anode in lithium–ion batteries

Authors: Filip Kozłowski [1], Daecheol Jeong [2], Beichen Xiong [2], Geoffrey Daniel [3], Rafał J. Wiglusz [4,5], Fredric G. Svensson [6], Juanjuan Lu [7], Haiyan Wang [7], Brian M. Tackett [2], Gulaim A. Seisenbaeva [1], Vilas G. Pol [2], Vadim G. Kessler [1,*]

[1] *Department of Molecular Sciences, BioCenter, Swedish University of Agricultural Sciences, Almas allé 5, Box 7015, SE-75007 Uppsala, Sweden*

[2] *Davidson School of Chemical Engineering, Purdue University, West Lafayette, IN, 47907 USA*

[3] *Department of Forest Biomaterials and Technology/Wood Science, Swedish University of Agricultural Sciences, Vallvägen 9C-D, 756 51 Uppsala, Sweden*

[4] *Division of Biomedical Physicochemistry, Institute of Low Temperature and Structure Research, Polish Academy of Sciences, Ul. Okolna 2, 50–422 Wroclaw, Poland*

[5] *Meinig School of Biomedical Engineering, College of Engineering, Cornell University, Ithaca, New York 14853–1801, United States*

[6] *Department of Materials Science and Engineering; Solid State Physic, Uppsala University, Ångströmlaboratoriet, Lägerhyddsvägen 1, Box 35, SE- 75103 Uppsala, Sweden*

[7] *Materials Engineering, Purdue University, West Lafayette, IN, 47907 USA*

Corresponding author: vadim.kessler@slu.se

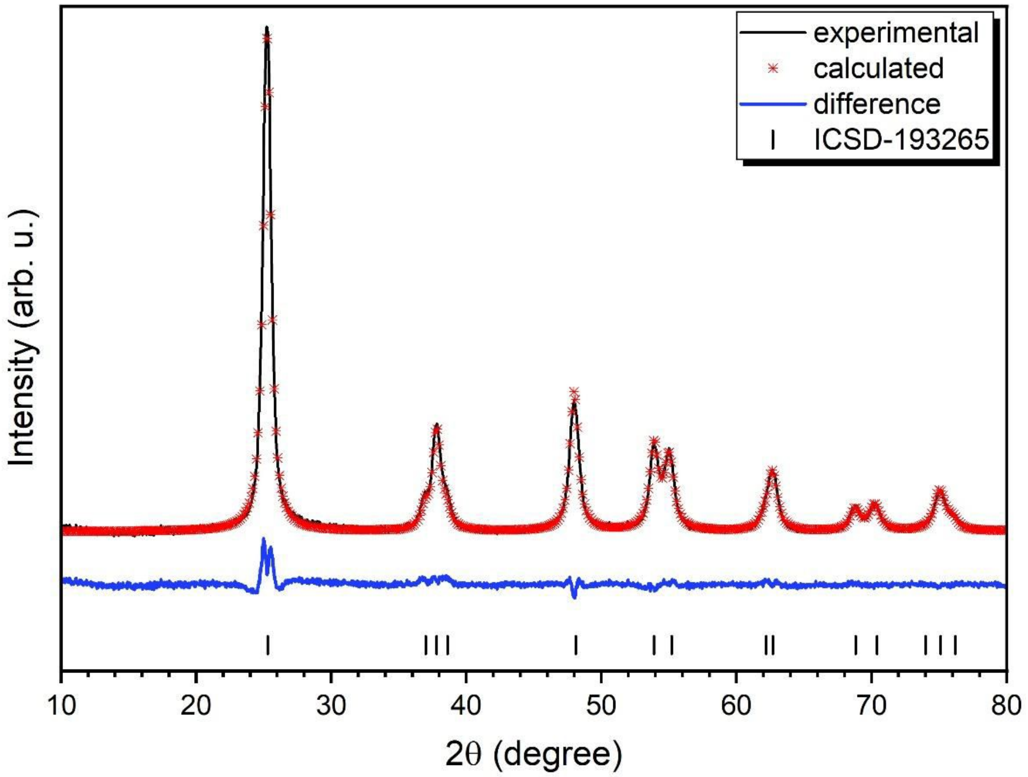

Figure S1. Representative results of the nanosized 2 mol% Mn^{4+} ion doped TiO_2 obtained at 500°C, Rietveld analysis (red–fitted diffraction; blue – differential pattern, column – reference phase peak position).

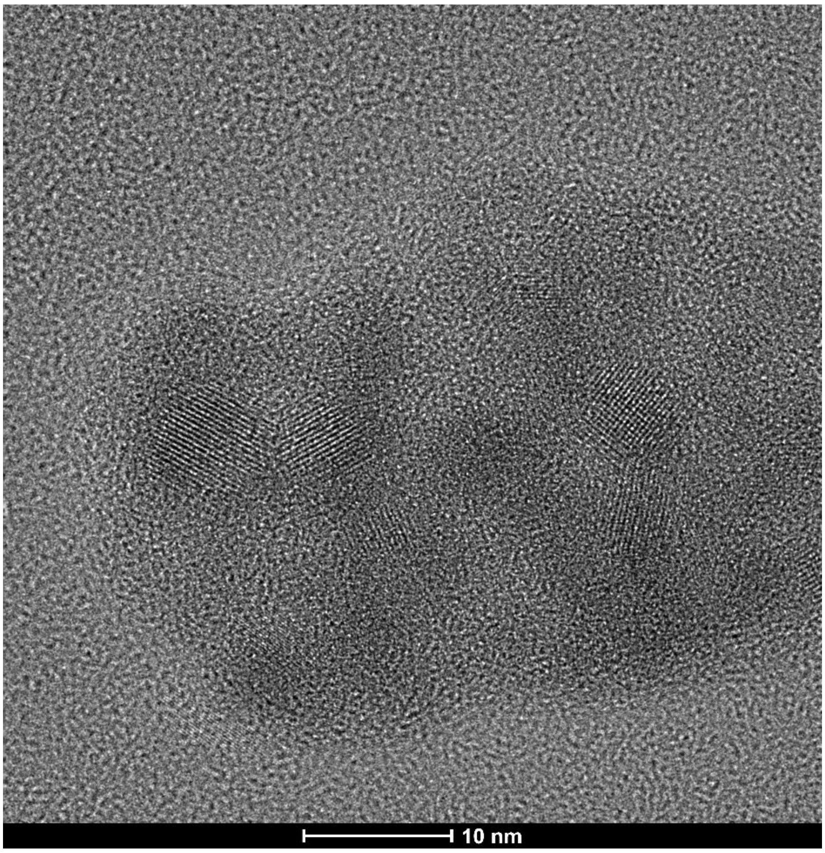

Figure S2. High resolution transmission electron microscopy (HRTEM) image of 20 mol% Mn-doped TiO_2 nanoparticles with high crystallinity.

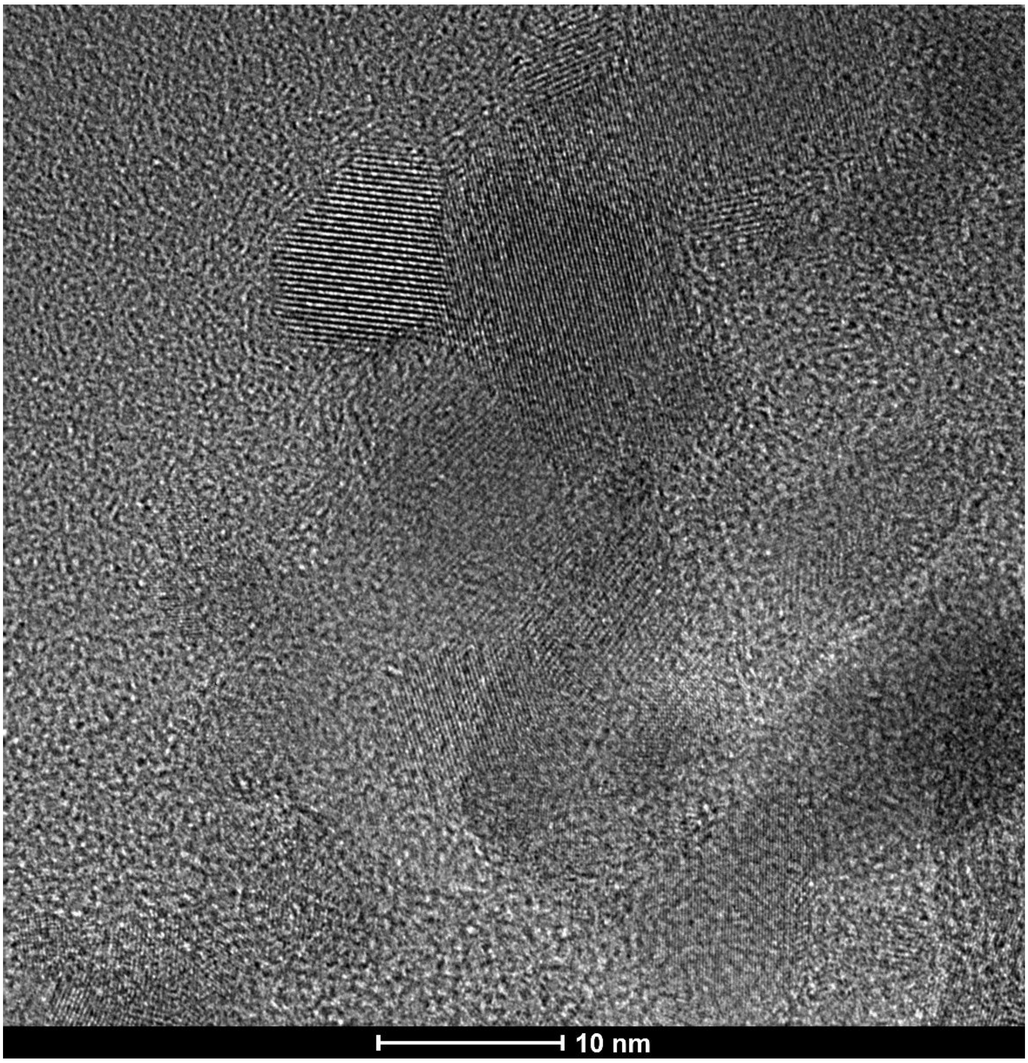

Figure S3. High resolution transmission electron microscopy (HRTEM) image of pristine TiO_2 nanoparticles with high crystallinity.

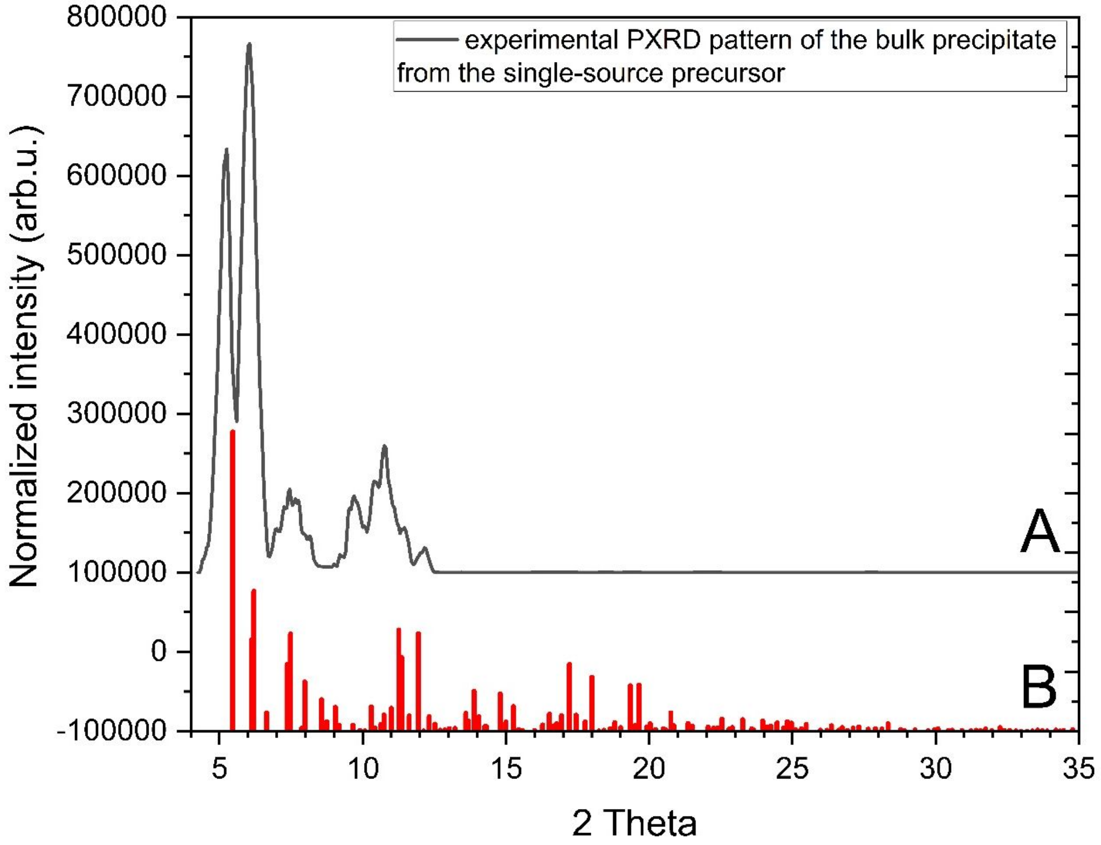

Figure S4. Simulated powder X-ray diffraction (PXRD) pattern, calculated from the single-crystal structure (B), overlaid with the experimental PXRD pattern of the bulk precipitate from the single-source precursor (A), exhibiting agreement and thereby validating the structural model and confirming phase purity.

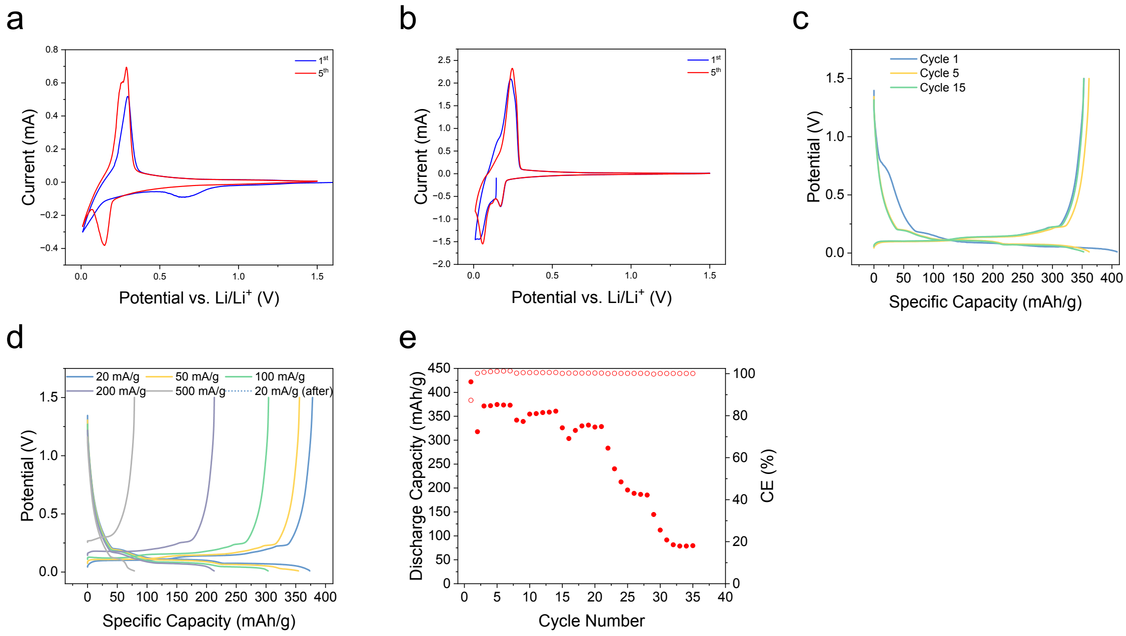

Figure S5. Electrochemical performance of Graphite. (a) and (b) Cyclic voltammetry (CV) of Graphite (a) the 1st cycle, showing SEI formation. (b) 1st and 5th cycle after formation, demonstrating a reversible electrochemical reaction. (c) and (d) Voltage–capacity profiles at various cycles for (c) Gr || Li cell at room temperature at 50 mA g^{-1} after five formation cycles at 20 mA/g. (d) Rate capability of graphite at various current densities. (e) Discharge capacity and Coulombic efficiency (CE) of the Gr electrode over rate cycling at room temperature.

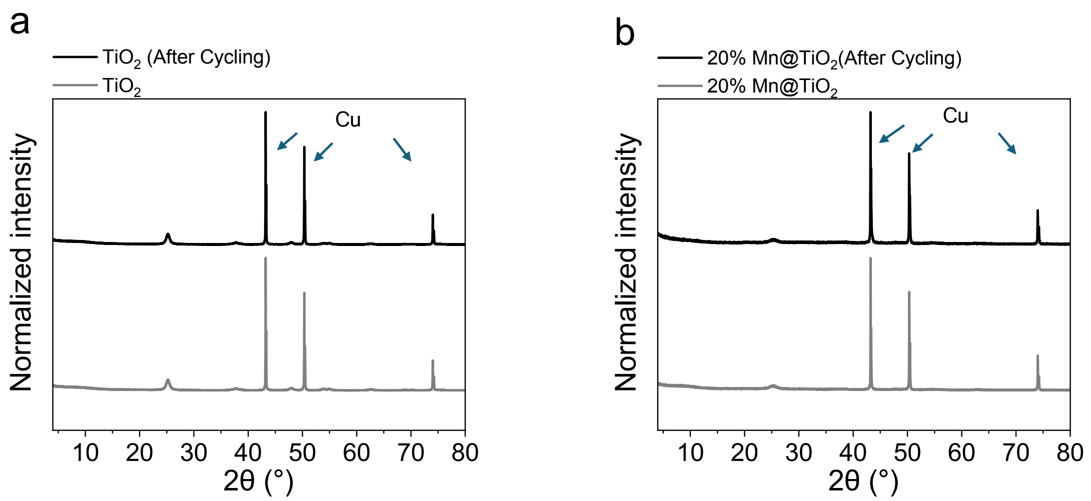

Figure S6. XRD patterns before cycling and after cycling. (a) of TiO_2 and (b) 20% Mn@TiO_2

Table S1. Comparison of unit cell parameters and phase composition for different samples. Unit cell parameters (a, c), crystal cell volume (V), and refinement factor (Rw) for nanosized materials, including x mol% Mn^{4+}:TiO_2, pure TiO_2 (anatase and rutile phases), MnO_2 and bixbyite (Mn_2O_3). Phase composition is presented as the percentage of anatase, rutile, MnO_2, and bixbyite phases determined from X-ray diffraction analysis.

Sample	***Cell parameters***											***Phase***				
	Anatase TiO_2			***Rutile TiO_2***			***MnO_2***			***Bixbyite M_2O_3***		***Anastase (%)***	***Rutile (%)***	***MnO_2 (%)***	***Bixbyite (%)***	***Rw***
	a (Å)	*c (Å)*	*V (Å³)*	*a (Å)*	*c (Å)*	*V (Å³)*	*a (Å)*	*c (Å)*	*V (Å³)*	*a (Å)*	*V (Å³)*	–	–	–	–	–
s. c.	3.781(9)	9.527(1)	136.2(6)	4.650(1)	2.969(7)	64.2(2)	4.397(0)	2.869(0)	55.4(7)	9.408(0)	832.7(1)	–	–	–	–	–
TiO_2, 500°C	3.787(9)	9.515(7)	136.53(3)	4.597(5)	2.961(2)	62.59(1)	–	–	–	–	–	83.40	16.60	–	–	2.00
TiO_2 hyd.	3.795(7)	9.474(2)	136.49(8)	4.650(1)	2.969(7)		–	–	–	–	–	99.67	0.33	–	–	2.62
x= 2, 500°C	3.790(1)	9.513(7)	136.66(3)	–	–	–	–	–	–	–	–	100	–	–	–	1.98
x= 2, 600°C	3.785(8)	9.515(9)	136.38(5)	4.593(5)	2.959(5)	62.44(6)	–	–	–	–	–	58.56	41.44	–	–	2.80
x= 2, 700°C	–	–	–	4.592(6)	2.959(4)	62.42(0)	–	–	–	–	–	–	100	–	–	2.44
x= 5, 500°C	3.790(4)	9.502(8)	136.52(8)	4.597(5)	2.947(7)	62.30(5)	–	–	–	–	–	97.94	2.06	–	–	1.95
x= 5, 600°C	3.786(0)	9.510(8)	136.32(6)	4.589(8)	2.957(6)	62.30(6)	–	–	–	–	–	21.85	78.15	–	–	1.41
x= 5, 700°C	3.787(5)	9.498(1)	136.25(2)	4.591(5)	2.959(0)	62.38(1)	–	–	–	9.419(7)	835.81(7)	5.17	93.53	–	1.30	2.32
x= 20, 500°C	3.794(5)	9.390(9)	135.21(2)	4.587(3)	2.950(3)	62.08(4)	–	–	–	–	–	66.45	33.55	–	–	2.00
x= 20, 600°C	3.787(0)	9.483(8)	136.01(1)	4.583(3)	2.954(9)	62.07(2)	–	–	–	9.408(0)	832.70(6)	3.03	89.10	–	7.87	2.90
x= 20, 700°C	–	–	–	4.591(1)	2.958(8)	62.36(6)				9.437(8)	840.64(4)	–	81.37	–	18.63	1.85
MnO_2	–	–	–	–	–	–	4.401(2)	2.874(7)	55.68(4)	–	–	–	–	100	–	2.90

s. c. – single crystal reference data, anatase– ICSD 193265, rutile – ICSD 190784, MnO_2 – ICSD 643197, bixbyite – ICSD 151411
